# An evaluation of a first-of-its-kind hybrid law degree program

**DOI:** 10.1007/s12528-022-09308-3

**Published:** 2022-02-23

**Authors:** Shuai Wang, Rebecca Griffiths, Claire Christensen, Cynthia D’Angelo, Kerry Condon

**Affiliations:** 1 School of Education, Shanghai Jiao Tong University, Shanghai, China; 2 Education Division, SRI International, Menlo Park, USA; 3 College of Education, University of Illinois at Urbana-Champaign, Champaign, USA; 4 Tyton Partners, Boston, USA

**Keywords:** Blended learning, Hybrid learning, Legal studies, Online learning, Education technology, Graduate education

## Abstract

There are few published studies investigating the effectiveness of hybrid formats at the program level in graduate legal education. A hybrid Juris Doctorate (J.D.) program launched by a Midwestern institution was the first ABA-accredited law degree program with a substantial online learning component. This study takes a mixed methods approach (both quantitative and qualitative) to evaluate student outcomes and the extent to which the hybrid program expands access to legal education. The study compares student outcomes in the hybrid program with full-time and part-time traditional, in-person programs at the same institution. After three terms of data collection, findings suggest that student outcomes and engagement are comparable across formats when controlling for student background characteristics and prior achievement. Evidence suggests that the hybrid option may increase access to legal education but is insufficient to determine whether the hybrid program will increase availability of legal services in underserved areas.

## Introduction

The global pandemic has created large amounts of uncertainty in higher education. Institutions and programs that have been willing and able to adapt and be flexible during this time are well-suited to meet their students’ and communities’ changing needs as we all navigate this new future. Hybrid and online instruction have gone from modalities that only a handful of programs use to a more mainstream and normalized way of delivering instruction, even if only for short periods of time. These alternative modalities have allowed students, faculty, and staff to continue learning in safer conditions. However, there is still a lot we do not know about how best to design and situate these hybrid programs and how to implement them effectively.

This article presents a comprehensive evaluation of the first hybrid law degree program accredited by the American Bar Association. Using a mixed-methods approach, this study compares the outcomes of a hybrid Juris Doctorate (J.D.) program to part-time and full-time traditional instruction at the same institution. The study contributes to the growing field of literature comparing online, in-person, and hybrid/blended learning formats. It constitutes the first evaluation of a hybrid Juris Doctorate program. It addresses a call for rigor and reliability (Lack, 2013) by reporting on a range of student outcomes, including multiple student academic outcome metrics and indicators of student engagement and experience.

The setting for this study is a Midwestern, practitioner-oriented law school located in a mostly urban area. It offers both full and part time degree programs and a number of certificate programs addressing high demand fields such as cybersecurity and health care. The institution employs approximately 170 full and part-time faculty and serves roughly 1,000 students, of whom just under half are enrolled full time.

## Defining ‘hybrid’

The educational research community lacks a definitive definition of hybrid learning. Scholars and practitioners frequently use hybrid interchangeably with blended learning to indicate a course delivery mode that mixes features of both online and in-person instruction. According to Reasons et al. (2005), “hybrid courses offer a combination of traditional and online teaching approaches, the intention being to provide the benefits of strategically timed class meetings coupled with the convenience of online learning activities” (p. 84). Twigg outlines a framework for categorizing types of hybrid/blended learning: replacement, supplemental, emporium, buffet (Twigg, 2003). In this study, the ‘hybrid’ or blended learning program can be categorized as replacement because the hybrid program combines intensive, week-long, on-campus sessions with periods during which coursework and lectures take place entirely online.

### Prevalence of hybrid/blended learning

The U.S. Department of Education reports that in fall 2018, 34.5% of all undergraduate students and 39.8% of all graduate students took at least one distance education course (NCES, 2019). Thirty-point-seven percent of all students enrolled in post-baccalaureate institutions exclusively took distance education courses in fall 2018 whereas roughly 14% of all undergraduate students participated in degree programs that were entirely online. More importantly, these proportions have all increased over time (NCES, 2014; NCES, 2016). These statistics indicate the prevalence of online learning as an option within postsecondary education, particularly at the graduate level.

In particular, the affordances of online technologies have played a crucial role in helping institutions and instructors respond to Covid-19-related disruptions to education, which caused 1.6 billion students to lose access to classrooms, in addition to numerous other challenges (UNESCO, 2020). As digital learning technologies continue to proliferate, there is a growing consensus that learning technologies will be integrated into existing educational systems (Al-Imarah & Shields, 2018; Aljawarneh, 2020; Pe´rez-Sanagustı´n et al., 2017; Reich & Ruipérez-Valiente, 2019). Chingos et al. (2014) outline trends toward both Massively Open Online Courses (MOOCs) and hybrid learning, and state that hybrid learning is a potentially optimal format for postsecondary education. Similarly, Shea et al. state that “hybrid courses, which combine face-to-face and online activities, are the fastest growing courses in higher education” (Shea, 2015, p.539).

### Comparisons of student outcomes across online and traditional formats

With the notable exception of several studies examining online courses in community colleges, researchers have generally found learning outcomes to be comparable across online and face-to-face (traditional) formats, all course and instructional aspects held constant (Larson & Sung, 2009; Nazar et al., 2019; Xu & Jaggars, 2014;). Several meta-analyses have found no difference or modest increases in student outcomes for online or hybrid courses versus traditional learning formats (Major et al., 2021; Schmid, 2014; Tallent-Runnels, 2006). These analyses additionally find that blended learning formats tend to produce slightly higher student achievement outcomes than either online or face-to-face courses alone (Asarta & Schmidt, 2020; Means, 2009; Schmid, 2014). Means et al. extensively review studies comparing learning formats, either traditional to online or blended to traditional or all three to one another (Means, 2009). On average, Means et al. find that blended learning conditions produce modestly higher student achievement results than face-to-face instruction. Bernard, et al. (2014) and Schmid et al. (2014), in their meta-analysis of blended learning studies and studies on technology in higher education generally, find a 0.3–0.4 higher effect size in terms of student achievement outcomes for hybrid/blended courses (Schmid, 2014). A recent review by Asarta and Schmidt likewise confirms better outcomes for blended courses; however, the authors find that the superiority of course formats varies by student prior achievement (Asarta & Schmidt, 2017).

Studies have also uncovered non-academic benefits of hybrid learning. Research similarly identifies hybrid courses as effective in enabling faculty to take advantage of accessible technology while continuing face-to-face interactions critical to student success (Oblender, 2002). Supporters also claim that these instructional formats can potentially serve a global market of professionals looking for flexible, lower-cost, alternatives to traditional professional degree programs (Joyner, 2018; Zheng, Chen, & Burgos, 2018). The positive outcomes associated with a hybrid/blended learning approach contribute to the growing popularity of this instructional format (Means, 2009; Schmid, 2014).

Hybrid/blended learning is increasingly a way that students are accessing post-secondary education. Understanding the ways in which traditional and hybrid programs are different and have different affordances for particular types of students, especially those in professional higher ed degree programs, is more important than ever, as more educational institutions are looking to hybrid and online approaches to supplement or replace their traditional offerings. Part of understanding this difference is understanding the many aspects of learning that are important for these types of programs.

### Theoretical framework: community of inquiry model

**Fig. 1:**
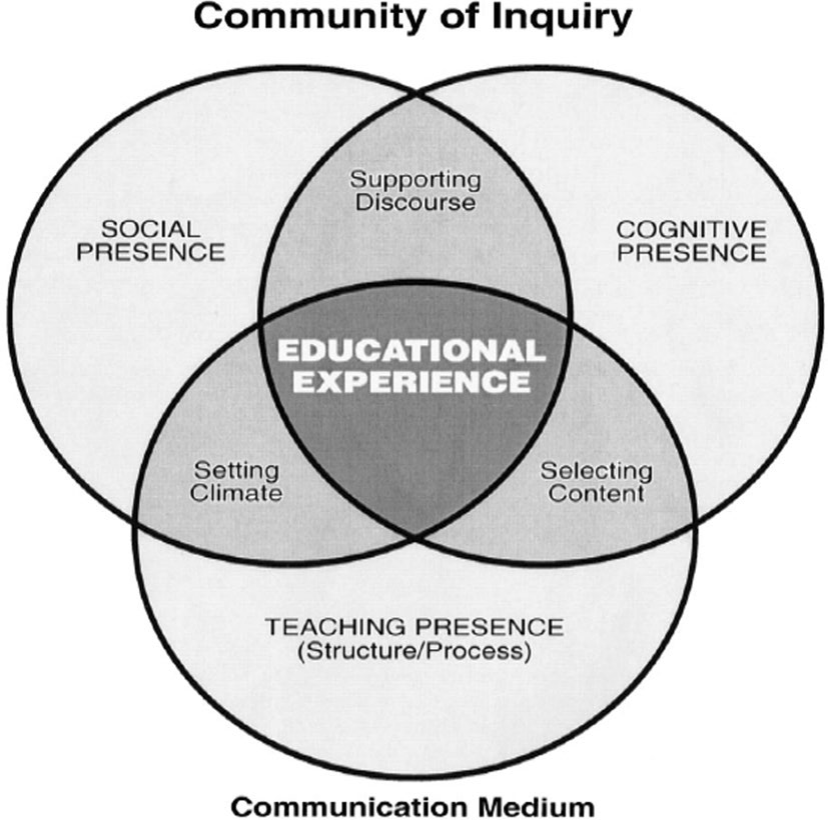
Elements of an educational experience (Garrison et al., 2000)

The Community of Inquiry (CoI) is a social constructivist theoretical model for learning in online courses (Castellanos-Reyes, 2020; Garrison et al., 2000). This model proposes that learning occurs within the community through the interaction of three core elements, including cognitive presence, social presence, and teaching presence (see Fig. 1). The CoI framework is grounded in the theory that students’ development of personal relationships with each other, identity in the class, and collective construction of knowledge are central to their learning (Garrison & Arbaugh, 2007; Garrison et al., 2000). These processes are facilitated by the environment and structures put in place by instructors, for example the extent to which students feel a sense of belonging and are invited to participate in class discourse (Anderson et al., 2001). The CoI has influenced scholarship on online learning over twenty years, including development and validation of instruments that measure components of CoI and critiques about its limitations, such as whether additional components are needed (Castellanos-Reyes, 2020). In the present study, elements of CoI theory were helpful in developing the student focus group and instructor interview protocols, which explored themes such as sense of community and belonging.

### Challenges in measuring student outcomes

Studies in postsecondary online learning frequently rely solely on course grades and cumulative GPA to determine student learning outcomes (Tallent-Runnels, 2006). Few studies of online formats can be generalized beyond a specific course (Bowen et al., 2014). Because faculty generally design their own assessments for course achievement, it is rare to have common measures of student outcomes to compare across courses or course sections. Such comparisons are further complicated by the instructor practice of curving grades by section. Specific to hybrid/blended learning, one study states that “the great majority of the empirical studies into blended learning are research interventions of short duration conducted at either the course or task level, focusing on just one or a few aspects of blended learning.” (Yuping et al., 2014).

However, prior studies apply different methods and strive for broader significance. For example, Xu and Jaggars use an instrumental variable approach and data on nearly 40,000 students to look at differences in student outcomes for online vs. face-to-face courses across multiple disciplines and student demographics (Xu, 2014). Xu and Jaggars generalize findings to the disciplines studied as well as the student population: community and technical college students. The present study seeks to continue in this vein by generalizing across programmatic courses, using common course assessments, retention rates and other metrics of student academic and engagement outcomes across three semesters and multiple courses.

#### Combining student academic outcomes and student experiences

To answer central questions of academic effectiveness, research in online and blended learning primarily usually focuses on student academic outcomes – course completion, grade point averages, and test results. However, using the CoI model it is critical to evaluate effectiveness beyond student achievement by considering measures of student engagement, impacts on student relationships with other students and faculty, and student and faculty impressions of skills and knowledge acquired when instructions offered shifted from the traditional space to the online space. These other, non-achievement focused, outcomes are very important to student success and need to be part of the evaluation in addition to more traditional outcomes in order to fully understand how these hybrid programs may differ from face-to-face instruction.

On average, students prefer hybrid/blended learning to traditional and online formats. According to a 2016 national survey conducted by EDUCAUSE, blended learning persists as the preferred modality among today’s postsecondary students (Brooks, 2016). A majority of today’s college students reported that they prefer and learn most from instruction offered in blended learning environments and identify technology as vital media to their academic success (Brooks, 2016). The survey notes an important change in the education field in which students are entering postsecondary institutions with an increasing comfort with technology and expectation of its use in courses (Brooks, 2016).

Studies typically measure students’ experiences of blended learning in higher education using surveys (Bliuc, 2007; Instructional Technology Council, 2018) such as end-of-course evaluations. However, these student satisfaction comparisons often fail to control for student background characteristics (Bowen, 2014; Shea, 2015). A unique strength of the present study is its comparison of student experiences using the Law School Survey of Student Experience (LSSSE), controlling for student background characteristics. The LSSSE is a large-scale, national survey that has been administered annually since 2004 and reached 196 law schools. This evaluation supplements LSSSE data with qualitative data from classroom observations, instructor interviews, and student focus groups in order to measure aspects of the CoI that are not typically included.

#### Statement of the problem

This study makes a novel contribution to the field by expanding the study of online education to a graduate law degree program. Evaluations of online education typically focus on undergraduate courses (Arbaugh, 2009; Asarta, 2017; Schmid, 2014). Fewer studies focus on online learning formats at the graduate level. Yet, graduate programs continue to expand their offerings through online and hybrid coursework (Tallent-Runnels, 2006). Public Affairs and Public Administration represent two disciplines of graduate education that have both significantly expanded and significantly attended to the evaluation of online coursework (Ginn & Hammond, 2012; Nollenberger, 2015; Shea, 2015). Similarly, nursing and medicine provide technologically advanced learning formats and conduct field-specific evaluations, particularly for graduate and professional training (Hugenholtz, 2008; Wilke, 2006; York, 2008). This study expands the field by evaluating the use of online education in a graduate-level program.

As Janus, Duhl, and Canick outline, legal education has maintained the same structure since its inception and is new to innovative delivery formats (Janus, 2014). Institutions may feel that legal education does not lend itself to hybrid learning because the field emphasizes learning legal practice through interaction with others. No other accredited law degree program had previously offered curriculum utilizing online instruction as a substantial component of a J.D. degree program.

While several studies have examined the adoption of legal education, they were mainly conducted at the undergraduate level and individual-course level (e.g., Caston & Hyam, 2017; Nazar et al., 2019). The current study examines the adoption of hybrid models of education within graduate legal education, which expands the current field by paying needed attention to a graduate-level degree program that blends online and face-to-face instruction in legal education (Means, 2009; Schmid, 2014). We used What Works Clearinghouse Standards (U.S. Department of Education, 2020) and Review Protocol for Using Technology to Support Postsecondary Student Learning Practice Guide Version 1.1 to support the development of research questions and selection of comparison variables. Specifically, we examined the differences between the hybrid program under the study and traditional-format program, regarding their implementation differences, student population differences, academic outcome differences, and perspective differences to provide a holistic view of the comparison.

#### Research questions

1. How is the hybrid program under study structured? What are the key differences from the traditional formats, both full-time and part-time?
2. Who enrolls in the hybrid program under study, and how do these students compare to those in traditional, campus-based programs?
3. Are students in the hybrid program achieving the desired learning objectives: specifically, how do their academic outcomes compare with those of students in the traditional programs?
4. What are student and instructor perspectives on the hybrid format?

## Methods

This study took a mixed-methods approach (both quantitative and qualitative) to investigate the research questions above. The quantitative analysis compares academic outcomes and survey results for students in three programs: hybrid, traditional, and part-time. The quantitative analysis demonstrates, using empirical data, the overall academic and experience differences across groups. Notably, students were not randomly assigned to the three groups because of their personal schedules (thus not experimental study), so student background variables were controlled in the statistical models to produce accurate results about the program differences in the quasi-experimental study, which can accommodate factors being studied over which researchers have no control (in this case, program assignment). The study also includes qualitative focus groups with students in the hybrid program, as well as interviews with faculty and administrators involved in the program, to provide detailed views about instructors’ and students’ perceptions about the hybrid program. The research team would like to provide a holistic view of the program using the mixed-method approach.

### Program types

The hybrid law program evaluated in this study takes place at a Midwestern institution located in a mostly urban area with an enrollment of nearly 1100 students. The institution established the hybrid program in order to better meet its mission of expanding access to legal education (Janus, 2014). The four-year hybrid program operates alongside a four-year part-time program and a traditional, 3-year full-time program. The hybrid program features predominantly online courses with in-person capstone experiences.

This study compares the hybrid program to two traditional, bricks-and-mortar J.D. programs at the same institution. One is a traditional, full-time, face-to-face J.D. program that takes three years to complete. Another is a part-time program in which students take face-to-face courses part-time in the evening or on weekends. Students have the option to switch between the full-time and part-time programs as needed. The researchers’ institution’s IRB has reviewed and approved the study.

### Program merger

During the study period, the school of law that is the subject of this study merged with another school of law. Administrators stated that the two programs had similar curricula and student bodies, and our analysis confirmed that students from the two schools did not differ on most background variables. To increase our statistical power for cross-program comparisons—and especially the size of our part-time comparison group—we therefore combined student data from both schools in our quantitative analyses and controlled for the background characteristics – age and gender—on which the schools did differ.

### Data sources

This report includes data from seven sources: administrative records, student focus groups, faculty and administrator interviews, course assessments, site visit observations, the Law School Survey of Student Engagement (LSSSE), and a student survey developed for this study. The study includes hybrid program students who enrolled during spring 2015 (Cohort 1), fall 2015 (Cohort 2) and fall 2016 (Cohort 3). Focus groups, interviews, and observations were conducted during two site visits during capstone weeks, one in fall 2015 and one in fall 2016. On both occasions, we interviewed Cohorts 1 and 2; Cohort 3 was neither interviewed nor observed. For the data collection timeline see Table 1. For details on the study sample refer to Table 2.

**Table 1:** Data collection timeline

Semester	Administrative records	Student focus groups	Faculty/administrator interviews	Course assessments	Site Visit observations	LSSSE	[*Blinded*] Student survey
Fall, 2015	1, 2	1, 2	✓	1	1, 2		
Spring, 2016	1, 2					1, 2	
Fall, 2016	1, 2, 3	1, 2	✓	3			1, 2, 3

Numbers indicate the student cohort for which data were collected: the study includes hybrid program students who *enrolled* during spring 2015 (Cohort 1), fall 2015 (Cohort 2) and fall 2016 (Cohort 3). Blank cells indicate that data were not collected. The research team conducted site visits to collect focus group, interview, and observation data in the fall 2015 and fall 2016 semesters. Focus groups were not conducted with Cohort 3 students because the timing of the site visit would interrupt their first on-campus week of instruction. We were unable to identify a common course assessment for Cohort 2

**Table 2:** Student samples

Program	Total students	WRAP scores available	Torts scores available	LSSSE data	Access survey responses
Hybrid	273	90	80	176	67
Traditional	522	144	44	340	N/A
Part-Time	176	67	1	117	N/A

### Administrative records

We obtained administrative records to investigate student academic outcomes. Following Lockee et al. (2001), we collected student academic outcomes as well as learner characteristics to understand program differences. The school shared de-identified administrative data pertaining to students’ academic outcomes and background characteristics. Academic data include students’ program designation (i.e., hybrid, traditional, or part-time), program start date, LSAT score, undergraduate grade point average (GPA), current law school GPA, years of education, and withdrawal status (e.g., whether and when the student withdrew from the program). Background characteristics include gender, date of birth, and race/ethnicity.

The school also provided financial information from students’ Free Application for Federal Student Aid (FAFSA) records. FAFSA is an application system by U.S. colleges, universities, and career schools for awarding federal, state, and college-funded student aid. Eligibility criteria include demonstrating financial need, being a U.S. citizen or an eligible noncitizen, etc. Filing a FAFSA is optional; some students in the sample did not file a FAFSA. The university provides eligible students with instructions and guidance about FAFSA application. To address these non-randomly missing data, we dichotomized the FAFSA data to indicate solely whether a student filed a FAFSA (i.e., 1 for filed, 0 for not filed). We use this as a rough indicator of students’ financial status, as those with higher incomes may be less likely to file a FAFSA.

### Student focus groups

We conducted student focus groups to investigate student experiences. We conducted site visits during two capstone weeks, in which hybrid students in Cohorts 1 and 2 visited campus for intensive learning activities. Focus group questions were open-ended and students were encouraged to voice differences of opinion. The purpose of these focus groups was to gain insight into students’ perceptions of the hybrid program, including their sense of belonging within the academic community, their identity, and ways in which the faculty may have supported community-building among the students and faculty; overall program strengths and weaknesses; career development supports; and how the program evolved over time. CoI was instrumental in developing the protocol. As an example, group cohesion and sense of belonging are important elements in social presence aspect of the CoI model, and in the student focus groups, we specifically sought information regarding student relationships with faculty (Thinking about other educational experiences you have had in the past: how would you compare your relationship with faculty in the hybrid program? What has helped you to develop relationships with faculty in the hybrid program?) and student relationships with other students (Thinking about other educational experiences you have had in the past: how would you compare your relationship with other students in the hybrid program? What has helped you to develop relationships with other students in the program?).

### Faculty and administrator interviews

We conducted faculty and administrator interviews to understand both student academic outcomes and student experiences. Interviews with faculty who taught both hybrid and traditional courses and administrators were conducted primarily on campus during site visits. A few interviews were conducted via phone after site visits. Interviews followed a semi-structured, open-ended format. Goals of the faculty and administrator interviews were to learn more about the structure of hybrid courses; perceptions of the types of students enrolled in the program and their course performance; relationships with students; and supports for student career development.

### Course assessments

We collected course assessment scores to investigate student academic outcomes. An important element of the evaluation was to identify a common post-test that could serve as a more fine-grained measure of student academic outcomes than course grades. We identified two assessments that were common to both hybrid and traditional students: the WRAP (Writing, Representation, Advocacy, and Problem solving) capstone memo in fall 2015 (Cohort 1) and the Torts final exam in fall 2016 (Cohort 3).

In fall 2015 the WRAP capstone memo assignment was identical across hybrid, traditional, and part-time programs. The capstone examination is a full office memo on a particular legal issue. It is graded by adjunct professors who are local practicing lawyers. The course coordinator trains all adjuncts on a common approach to grading, and all adjuncts grade students on a common rubric. Curving takes place at the course level, rather than the adjunct level, so grades are comparable within the same course.

In fall 2016, we compared hybrid and traditional students’ grades on the Torts final examination because the WRAP capstone memo was no longer common to both hybrid and traditional students. All questions were identical for both hybrid and traditional students, and all were graded by the same professor. The school provided students’ un-curved exam letter grades for analyses. These were matched with other datasets and de-identified before being sent to the research team.

### LSSSE data

We collected LSSSE data to investigate student experiences. The Law School Survey of Student Experience (LSSSE), a widely used and validated survey of student engagement, was administered in spring 2016 to all J.D. students at the law school (Silver, 2012). To analyze student LSSSE outcomes across the three program formats, we matched LSSSE data with administrative data. At the authors’ request, LSSSE added several items for this study regarding where students plan to live and practice law.

Our analyses examined LSSSE Engagement Indicators, a set of scales developed by LSSSE measuring distinct aspects of student engagement (LSSSE, 2017). Each indicator combines multiple questions that address different aspects of a common theme, together providing a more complete measurement of that overall construct. LSSSE Engagement Indicators include: learning to think like a lawyer; student-faculty interaction; student advising; and overall environment of a law school (LSSSE, 2017). The learning to think like a lawyer indicator explores the extent to which law school courses emphasize various critical thinking activities and how students experience their intellectual growth. The student-faculty interaction indicator assesses students’ perceptions of their interactions with faculty related to both academic and non-academic matters. The student advising indicator includes questions about advisory services offered by law schools. The overall environment of a law school indicator measures students’ perception of the law school environment and their ‘fit’ in that environment.

### Access survey

To explore whether the hybrid program has the potential to expand access to legal education, we designed and conducted a voluntary student survey. Items asked about students’ access to legal education, current field of work, and reason for choosing the hybrid program. The survey was sent to all students in the hybrid program; of them, 69 students entered the survey and 67 consented to participate. See total number of students by program in Table 2.

### Mixed-method analytic approaches

Both qualitative and quantitative examinations were conducted to gain a holistic view of the program.

### Qualitative analysis

We conducted qualitative analyses of notes from site visit observations, student focus groups, and faculty and administrator interviews to inform our understanding about student experience. Interview protocols were informed by the Community of Inquiry framework, which is widely used in online learning research (Garrison, 2000). This framework examines the development of three crucial and inter-related elements of a meaningful online learning experience – social, cognitive, and teaching presence (Garrison, 2000).

The coding scheme was co-developed by two of the authors who went through an iterative process of code refinement that emerged from the collected qualitative data. Transcripts were first coded broadly for their applicability to each of the evaluation’s research questions. Researchers then identified sub-codes within each research question. Sub-codes emerged from the focus group and interview data and were intended to represent a range of experiences associated with the hybrid program. The authors discussed and resolved the disagreements during the coding process.

#### Quantitative analysis

We first compared students’ background characteristics among the hybrid, part-time and traditional programs. Linear regression models were used to examine continuous variables (age and years of education), while logistic regression was performed for binary categorical variables (female, minority, and filed FAFSA). All subsequent analyses were conducted with linear regression and logistic regression controlling for students’ background characteristics which varied significantly across the three groups, and those that were significantly related to the dependent variables.

In summary, through mixed method (both qualitative and quantitative analyses), we compared hybrid, part-time traditional, and full-time traditional students on the following academic outcomes and student experiences:

- Academic Outcomes:
  o Law school GPA
  o Withdrawal rates
  o WRAP Memo
  o Torts exam scores
- Student Experiences:
  o Four LSSSE indicators of student engagement.
  o Student focus groups
  o Faculty and administrator interviews

## Results

### How is the hybrid program under study structured? What are the key differences from the traditional formats, both full-time and part-time?

The hybrid J.D. program is a 4-year, part-time program. For the majority of the semester courses are taught online. Delivering instruction online also involved relevant changes to instructional approaches. Specifically, students come to campus for one week toward the end of each semester, known as Capstone Week, for live instruction and intensive case simulations. Students also come to campus for an orientation period during the first week of their first semester in the program.

Many of the program course structure elements were the same, including course content, materials, and assessments; the availability of online course electives; and faculty office hours. The hybrid program includes some unique elements, including Capstone Week and graded discussion board posts. Hybrid courses are more likely to employ adjunct faculty for grading purposes than are traditional courses.

#### Course structure

Professors interviewed in fall 2016 indicated that they were encouraged to use the same textbooks, assignments, and rubrics in both traditional and hybrid courses. Course pacing differs though —for example, to accommodate Capstone Week.

Hybrid and traditional courses differ on delivery mode. Traditional program students participate in weekly live lectures, whereas hybrid students view lectures asynchronously via video. Professors in the hybrid program typically post at least one video lecture per week. Video lectures typically include a PowerPoint presentation and professor narration. Rather than engaging in live class discussion, students in the hybrid program are graded on their posts and replies in online discussion boards. Much like in a traditional program, faculty in the hybrid program offer online, synchronous office hours via video chat. All hybrid program instructors were encouraged to follow the same course design consistently across courses and semesters, including video lectures, discussion boards, and synchronous office hours. Instructors’ implementation of these elements could vary. These elements are further described in Table 3.

**Table 3:** Common elements in hybrid courses

Element	Description	Professors mentioning
Video lectures	Lectures typically include a PowerPoint and professor narration. Professors typically post at least one lecture per week. Reported lecture length ranged from 5 to 90 min. Students watch lectures asynchronously	6
Discussion board posts	Students are graded on their discussion board posts and replies. The number of required discussion posts reported per course ranged from 1 to 3 per week	6
Office hours	Office hours are synchronous video conversations via Blackboard Collaborate, typically offered weekly. To maintain flexibility for working students, attendance at office hours is not required or graded, and office hours are recorded for asynchronous viewing. Instructor-reported attendance at office hours ranged from 1 to 30 students, out of about 95 students per class	5

Professor response rates are out of six professors responding to interview questions

Online elective courses are offered to both hybrid and traditional students, with hybrid students receiving priority registration. In fall 2016 the program offered its first two elective courses. The program also plans to add a specialized track in health law in response to student requests.

#### Capstone week

During Capstone Week students participate in case simulations designed to resemble the practice of law. In a typical Capstone Week simulation, students must work as teams of attorneys to resolve a legal case. Faculty interact with students in character as clients, senior attorneys, and judges, assessing students and providing feedback throughout. Activities are designed to elicit and assess a range of lawyering skills including document review, letter drafting, fact gathering, negotiating, and contract revision.

#### Adjunct instructors

Because hybrid students are assessed more frequently than traditional students, the hybrid program has a greater grading burden. To meet this demand, the hybrid program hires adjunct professors to grade assignments, while full-time professors establish the curriculum and provide most of the course instruction. This division of labor is unique; in traditional courses at this institution, full-time professors are typically responsible for both instruction and grading.

Adjunct instructors are practicing local lawyers. According to an administrator, hiring priorities for hybrid-program adjuncts include practice experience (typically 3–7 years), subject matter knowledge, and interest in connecting with students. An administrator reported that adjuncts participate in two mandatory training workshops each semester: one about technology use, and one about best practices when grading and teaching online. The program also provides an adjunct manual, outlining expectations for grading turn-around and appropriate feedback. Course professors oversee adjuncts’ work—for example by explaining grading criteria and investigating cross-adjunct grading consistency.

### Who enrolls in the hybrid program under study, and how do these students compare to those in traditional, campus-based programs?

Compared with traditional, full-time students, students in the hybrid program tended to have lower undergraduate GPAs, to be older, and to have more years of education. Instructors noted that hybrid students are more likely to have established careers, which enriches course participation.

#### Comparison of prior achievement

We analyzed undergraduate GPA and LSAT scores as indicators of students’ prior achievement. Table 4 presents means and standard deviations, along with statistical significance. Although values varied among the 3 groups, regression results showed that most of the differences were not statistically different, including differences in average LSAT scores (*F* [2, 967] = 0.59, *p* = 0.56). Traditional students had higher average undergraduate GPAs than students did in the other two programs (*F* [2, 907] = 5.72, *p* < 0.01). The degrees of freedom differed for different variables because of missing values.

**Table 4:** Prior achievement for hybrid, part-time, and traditional students

	Mean (SD)		
	Hybrid	Part-Time	Traditional
Undergraduate GPA	3.14 (0.44)	3.18 (0.46)	3.25 (0.40)**
LSAT score	151.77 (5.98)	151.14 (6.59)	151.59 (6.10)

^**^p < .01

#### Comparison of student background characteristics

We compared background characteristics among hybrid, part-time, and traditional students. Table 5 presents means and standard deviations (SD), along with statistical significance. Regression results showed that students in the hybrid program were on average older (*F* [2, 968] = 265.86, *p* < 0.001) and had more years of education than students in the other two programs (*F* [2, 959] = 65.27, *p* < 0.001).

**Table 5:** Background characteristics for hybrid, part-time, and traditional students

	Mean (SD)		
	Hybrid	Part-Time	Traditional
Age	40 (9.50)	33 (6.87)**	28 (5.82)**
Years of education	16.83 (1.24)	16.33 (0.80)**	16.11 (0.54)**

	Percent		
% Female	50.2%	50.6%	52.8%
% Minority	19.4%	22.7%	25.1%
% Filed FAFSA	70.0%	77.3%	78.9%

^**^p < .01

#### Comparison of hybrid students among three cohorts

Students’ prior achievement and background characteristics were examined among 3 cohorts of hybrid students (spring 2015, fall 2015, and fall 2016). Table 6 presents the descriptive statistics and statistical analysis results for these characteristics. Regression results indicated that fall 2016 hybrid students had lower (marginally significant) average undergraduate GPAs than spring and fall 2015 hybrid students (*F* [2, 244] = 2.75, *p* = 0.066). Logistic regression showed that fall 2016 hybrid students were more likely to file FAFSA applications than were spring 2015 hybrid students (χ^2^(1, N = 273) = 3.95, *p* = 0.05.).

**Table 6:** Prior achievements and background characteristics for three cohorts of hybrid students

	Mean (SD)		
	Spring 2015	Fall 2015	Fall 2016
Undergraduate GPA	3.20 (0.43)	3.17 (0.45)	3.05 (0.44)*
LSAT score	152.29 (6.24)	152.25 (5.60)	150.88 (6.06)
Age	40.57 (9.89)	40.70 (9.72)	39.73 (9.02)
Years of education	16.75 (1.21)	16.88 (1.32)	16.15 (3.56)

	Percent		
% Female	46.25%	51.04%	52.58%
% Minority	18.75%	20.83%	18.56%
% Filed FAFSA	62.5%	68.75%	77.32%*

^*^p < .05

#### Instructor impressions of hybrid students

Instructors stated that hybrid students have established careers and that this diverse life experience enriches class discussions and coursework. Some instructors felt that this experience makes students more engaged in courses and in discussions.

Instructors expressed some concern about declining admissions standards in both the hybrid and traditional programs. The administrative data do suggest a trend towards slightly lower undergraduate GPAs and LSAT scores for hybrid students, but so far differences are modest, and there has been no statistically significant decline in successive cohorts’ LSAT scores.

#### Relation of current job to legal education

Some students enroll in the hybrid program to further their current careers in legal fields or in businesses with legal components. For example, one student is currently working in insurance and plans to move into underwriting upon graduation. Others currently work in unrelated fields, often because they deferred their legal aspirations for practical reasons. Some of these students plan to move to a legal career upon graduation; some do not.

**Fig. 2:**
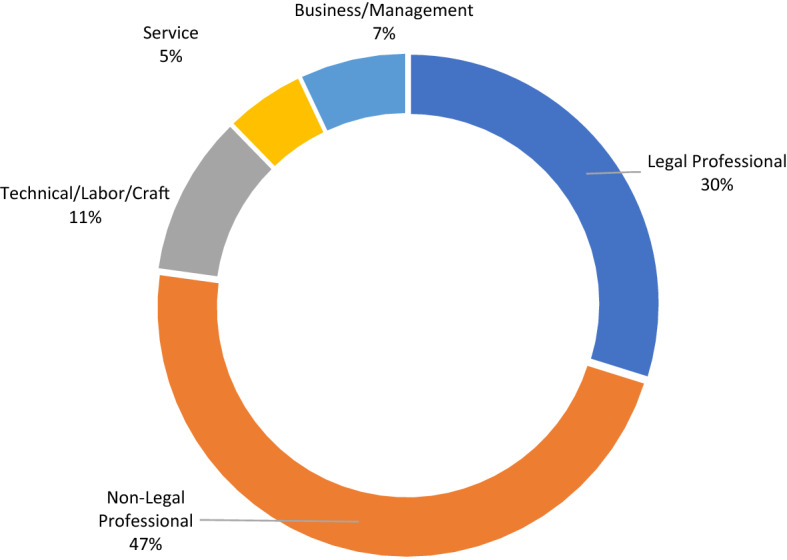
Student current career

Our student access survey asked students about their current field of employment. Figure 2 displays their responses. Thirty percent of respondents are using the hybrid law program to advance careers within the legal field. Nearly half of respondents are employed in non-legal professional fields such as medicine, education, and information technology. These students may be using the hybrid program to advance their careers in these fields, or to switch careers. About 16% of respondents indicated that they worked in technical, labor, craft, or service fields. These students may be using the hybrid program to make a more dramatic career transition.

Surprisingly, when we examined LSSSE data on hours worked outside of class, we did not see substantial differences between the three programs. This may be in part due to relatively low response rates for these items.

### Are students in the hybrid program achieving the desired learning objectives? How do their academic outcomes compare with those of students in the traditional programs?

In this section, we compare academic outcomes for students in the hybrid program with those of the other two formats. Academic outcomes include course assessments (WRAP capstone memo and Torts final exam), cumulative grade point average (GPA), and retention data. We also present qualitative data on student achievement in writing, practical skills and preparation for the Bar exam. We found that hybrid students did not differ significantly from students in the traditional programs in terms of law school GPA, retention rate, or WRAP capstone memo score. Students in the traditional, full-time program scored higher on the Torts final exam than those in the hybrid program.

#### Law school grade point average

We compared cumulative law school GPA among hybrid, part-time, and traditional students. Our analyses adjusted for the effects of students’ undergraduate GPA, age, and years of education because these variables varied significantly among the three student groups. We also controlled for LSAT and minority status in the model because these characteristics were significantly related to the Law GPA. We did not detect a significant difference in law school GPA among the three student groups. See Table 7 for descriptive statistics and Table 8 for regression results.

**Table 7:** Outcome measures for hybrid, part-time, and full-time students

	Hybrid	Part-time	Full-time
WRAP capstone memo score (Cohort 1)	41.1 (7.1)	39.6 (6.8)	39.1 (8.6)
Torts final exam score (Cohort 3)	8.11 (1.69)	NA	8.68 (1.62)*
Withdrawal rate^a^	7.96%	2.27%	2.87%
Current GPA	3.01 (0.57)	2.99 (0.70)	3.01 (0.53)

Means and standard deviations are reported for final exam score and current GPA. Percentages are reported for withdrawal rate

^a^This analysis excludes students from one school of law, because withdrawal rates were not available from this institution

^*^A statistically significant difference from the hybrid program

**Table 8:** Regression results for law GPA

Model description	Estimate	SE	*t*	*p*
Intercept	−3.76	0.44	−8.55	< .001
Part Time (Reference = Hybrid)	0.01	0.05	0.17	.86
Traditional (Reference = Hybrid)	−0.02	0.04	−0.54	.59
Undergrad GPA	0.30	0.03	8.78	< .001
LSAT	0.04	0.00	15.20	< .001
Age	0.00	0.00	0.50	.62
Years of Edu	0.01	0.01	0.87	.38
Minority (Reference = Non-Minority)	−0.11	0.04	−3.08	< .01

#### Retention

We compared retention rates among hybrid, part-time, and full-time traditional students. Our analyses adjusted for the effects of students’ undergraduate GPA, age, and years of education, because these variables significantly varied among the three student groups. We controlled for LSAT, an indicator for student prior achievement. We also controlled for FAFSA filed or not in the model because it was significantly related to the retention. As shown in Table 7, hybrid students had a higher withdrawal rate than students in the other two formats; however, this difference was not statistically significant when we controlled for background characteristics. See Table 9 for logistic regression results.

**Table 9:** Logistic regression results for withdrawal rate

Model description	Estimate	SE	*χ2*	*p*
Intercept	2.68	5.04	0.28	.59
Part Time (Reference = Hybrid)	−0.47	0.42	1.27	.26
Traditional (Reference = Hybrid)	−0.04	0.32	0.02	.89
Undergrad GPA	0.20	0.44	0.20	.65
LSAT	−0.04	0.03	1.90	.17
Age	0.03	0.02	1.58	.21
Years of Edu	−0.03	0.08	0.17	.68
FAFSA Not Filed (Reference = Filed)	1.05	0.20	27.41	< .001

Furthermore, when we look term by term, the number of withdrawals by cohort has declined over time: in the first cohort, 11 students dropped within the first two semesters of the program, while only 8 students withdrew from the second cohort over the same period of time. The fall 2016 cohort had only had one student withdraw during the first term.

#### WRAP capstone memo scores

We compared WRAP capstone memo scores for fall 2015 among hybrid, part-time, and traditional students. This type of course level assessment has an advantage over cumulative GPA, because scores on this assessment were not curved and thus are more directly comparable. Our analyses adjust for the effects of students’ age, years of education, undergraduate GPA, LSAT score, and gender because they were significantly associated with the WRAP capstone memo score and/or with pre-defined differences between student groups.

Because WRAP capstone memos were graded by different adjunct instructors, WRAP analyses modeled the nesting of students within adjuncts (ICC = 0.45). The results of hierarchical linear modeling indicated that there was no significant difference among the three groups of students (see Table 7 and Table 10). It is important to note, however, that these data represent final grades for only one semester of WRAP.

**Table 10:** Hierarchical linear modeling results for wrap memo

Model description	Estimate	SE	*t*	*p*
Fixed effects				
Part time (Reference = Hybrid)	−0.51	1.42	−0.36	.72
Traditional (Reference = Hybrid)	−0.98	1.37	−0.71	.48
Undergrad GPA	3.22	1.03	3.12	< .01
LSAT	0.40	0.08	5.29	< .001
Age	0.16	0.07	2.37	.03
Years of Edu	−1.24	0.54	−2.28	.03
Random effects				
Level-1 Intercept	−16.12	14.64	−1.10	.27
Level- 2 Intercept	24.54	7.67	3.2	< .001

#### Torts final exam

We compared Torts exam scores between hybrid and full-time, traditional students. Part-time students were excluded from the analysis because only one student from this group participated in the Torts exam. Our analyses adjusted for the effects of students’ undergraduate GPA, age, and years of education because these attributes varied significantly among the three student groups. We also controlled for LSAT and FAFSA filed or not in the model because they were significantly related to the Torts scores. Traditional students had significantly higher scores than hybrid law student groups (see Table 7 and Table 11). As noted earlier, instruction in the hybrid and traditional course formats addressed different competencies, so differences in assessment scores may reflect different emphases in the class focus.

**Table 11:** Regression results for torts final exam

Model description	Estimate	SE	*t*	*p*
Intercept	−14.56	3.81	−3.82	< .001
Traditional (Reference = Hybrid)	0.94	0.38	2.45	.02
Undergrad GPA	0.90	0.34	2.62	.01
LSAT	0.12	0.02	5.30	< .001
Age	0.03	0.02	1.93	.06
Years of Edu	−0.01	0.06	−0.22	.83
FAFSA Not Filed (Reference = Filed)	0.85	0.36	2.36	.02

The hybrid J.D. program delivers similar academic content as traditional programs at the same institution. It differs in that students watch lectures via video; write weekly graded discussion board posts; and attend one intensive, in-person Capstone Week each semester. Students in the hybrid program tend to have lower undergraduate GPAs, to be older, and to have more years of education than students in the traditional program. They report that the hybrid program expands their access to legal education by virtue of its compatibility with their work and family responsibilities. Students in the hybrid program do not differ from those in traditional programs on most programmatic measures of academic achievement. Likewise, their LSSSE experience ratings do not differ from those of students in the traditional programs.

### What are the student and instructor perspectives on the hybrid format?

Students in the hybrid program did not differ from those in the part-time or full-time traditional programs in terms of their program experience ratings on the Law School Survey of Student Engagement. Student and faculty-reported strengths of the hybrid program included pedagogy, flexibility, and institutional reputation; workload was noted as a primary challenge.

#### Comparison of LSSSE responses

[*Removed for blinded review*] used LSSSE data to compare students in the hybrid, traditional full-time, and traditional part-time programs on four scales designed to measure the student experience: learning to think like a lawyer, student-faculty interaction, student advising, and overall environment of a law school. All comparisons control for undergraduate GPA, age, and years of education, because these attributes varied significantly among the 3 student groups. Groups did not differ in how they rated their program on learning to think like a lawyer (*F*(2, 259) = 2.87, *p* = 0.06); student-faculty interaction (*F*(2, 263) = 1.27, *p* = 0.28); student advising (*F*(2, 189) = 1.92, *p* = 0.15); or their impressions of the overall law school environment (*F*(2, 249) = 0.01, *p* = 0.99). These findings may reassure those who worry that the large online component of the hybrid program substantially diminishes the student experience.

#### Student and faculty impressions of the hybrid program

In interviews and focus groups, students and instructors indicated that strengths of the hybrid program include:

- **Pedagogy:** Reported educational strengths of the hybrid program include course design and frequent assessments. Hybrid courses center on learning outcomes, with activities, materials, and assessments aligned to these outcomes.
- **Flexibility:** Students praised the flexibility of the program to fit their schedule and cited flexibility as one of the key benefits of the hybrid program. For example, the use of recorded lectures allows students to consume these at their preferred time and pacing. A student said:
  I couldn’t be with my family every night if I had to go to law school… I started my son was five months old. I would have missed so many of his firsts, so that flexibility is definitely number one.
- **Emphasis on writing and frequent assessments:** The hybrid program substitutes frequent written assignments for in-class discussion during the online portion of the term. These assignments allow students to receive frequent feedback, and many students and professors stated that the hybrid program builds strong writing skills due to the amount of practice. These frequent assignments also promote student engagement. Students said they appreciated that they were actively engaged and receiving feedback throughout the semester. Weekly deadlines required them to keep on top of their coursework and better prepared them for final exams.
- **Student–student relationships:** Students reported that the atmosphere among hybrid students is more collaborative than competitive, in part because they are from diverse geographical regions and thus are not competing in the same job market. Some students felt that the sense of community among students in this program is stronger than in other online programs.
- **Student-instructor relationships:** Students reported positive relationships with faculty, and instructors also felt that they had good relationships with students. Some students felt that their relationships with hybrid faculty were better than in other online learning programs. For instance, a student said:

I actually did online education before and I couldn’t tell you a single professor’s name. I never had a conversation with any of them… I feel like it’s completely the opposite here. I feel like they’re making a good effort to try to get to know, at least to some extent, every single student in the program.

Students also described some common needs: manageable workload, workload coordination across classes, and technical support access across varying time zones. Instructors described efforts to improve coordination among classes to make the workload more manageable. Instructors also noted that it is important for a hybrid program to create opportunities for students to practice verbal as well as written communication skills. Based on formative feedback, the program made efforts to help students manage the workload and to strengthen technical support.

## Discussion

This study is a mixed-methods, quasi-experimental evaluation of a hybrid J.D. program. Online learning is a popular postsecondary education option, particularly among graduate students (NCES, 2014). Meta-analyses suggest that students receiving instruction in online courses on average have similar or slightly better outcomes relative to those in traditional courses formats (Schmid, 2014; Tallent-Runnels, 2006; Ward, 2004). Yet few studies focus on online or hybrid instruction at the graduate level, fewer still use measures that generalize beyond a single course (Bowen et al., 2014), and none to our knowledge have evaluated hybrid legal education programs. This study breaks new ground in these respects.

Initial results suggest that a hybrid format can expand access to legal education without compromising quality. It does so, in part, by supplementing the online learning experience with virtual instructor office hours, weekly graded discussion posts, and intensive in-person capstone week. Consistent with meta-analyses of hybrid/blended learning outcomes (i.e., Schmid, 2014; Tallent-Runnels, 2006; Ward, 2004), this study finds minimal differences between program formats with respect to student achievement and engagement.

These findings are noteworthy for two reasons. First, we used two un-curved course assessments and retention rate as measures of academic achievement, which strengthen our ability to generalize students’ performance at the program level. In addition, comparisons of student engagement measures control for student background characteristics, reducing the threat of self-selection bias in our findings.

The hybrid program appeals to students who feel that traditional legal education programs are not compatible with their work or family responsibilities. Consistent with this finding, students in the hybrid program tend to be older and to have more years of education than those in the traditional part-time or full-time programs. Their maturity and experience may contribute to their success in the hybrid program as well as enhancing class discussions.

The Community of Inquiry model proposes that success in these hybrid programs relies on three elements: cognitive presence, social presence, and teaching presence. Students in this hybrid program reported a stronger sense of belonging and student to student community than other programs, contributing to the social presence in their academic community. The increased flexibility in these programs allows students to access course content when it fits their schedule, which can afford more of a cognitive presence when engaging with it. The frequent written discussions and alternate ways of engaging with faculty members contributed to an increased teaching presence for the students. Taken together, all of the design decisions of the hybrid program contributed to successful implementation of the curriculum. Hybrid programs should be designed to take advantage of the affordances of a hybrid model to expand access to graduate programs.

### Limitations

The primary limitation of this evaluation is that students self-select into different conditions. While we control for observable characteristics, we cannot rule out the possibility that students in one condition are more motivated or otherwise likely to succeed. For all analyses, it is important to keep in mind that the lack of statistically significant differences does not mean that the formats are necessarily equivalent; it is possible that our sample size is too small to be able to detect differences. Comparisons of the Torts and WRAP assessments are limited by the fact that we were unable to arrange for blind grading, so it is possible that instructors who graded the assignments were influenced by preconceptions about the performance of students in different programs. Additionally, we cannot be sure that filing or not filing FAFSA is a strong indicator of income status. Finally, participation in the two surveys (LSSSE and student access survey) was voluntary, so the sample may be biased. It may be that students who felt especially satisfied or dissatisfied with the hybrid program were more likely to respond. It is also possible that hybrid students felt induced to report positively about the program to increase its credibility in the legal community, or that their relatively high ratings for some indicators are based on different expectations about the experience in a hybrid program.

While the data sources and analyses have limitations, the findings generally suggest that, as of fall 2016 (i.e., two years into the program for the first cohort), there are not significant differences in outcomes among hybrid students compared to other formats. The combination of analyses gives us greater confidence that the hybrid format so far is comparable to the traditional format in terms of effectiveness.

### Recommendations

Given the study limitations, it would be valuable to track hybrid student cohorts through program completion and bar examination to increase confidence that the hybrid format effectively prepares students for future legal professions. Additionally, the question of whether hybrid programs can increase the availability of legal services in underserved areas, particularly in rural areas, merits further investigation.

Legal education may be a fruitful arena for further research in advanced postsecondary education given its common summative assessment: the bar exam. As online and hybrid education expands in postgraduate degree programs, it is vital to understand differences in program structures and their potential to affect student access, learning, and degree completion.

## References

[CR1] Al-Imarah AA, Shields R (2018). MOOCs, disruptive innovation and the future of higher education: A conceptual analysis. Innovations in Education and Teaching International.

[CR2] Aljawarneh SA (2020). Reviewing and exploring innovative ubiquitous learning tools in higher education. Journal of Computing in Higher Education.

[CR3] Anderson, T., Rourke, L., Garrison, D. R., & Archer, W. (2001). Assessing teaching presence in a computer conferencing environment. *Journal of Asynchronous Learning Networks*, *5*(2).

[CR4] Arbaugh JB, Godfrey MR, Johnson M, Pollack BL, Niendorf B, Wresch W (2009). Research in online and blended learning in the business disciplines: Key findings and possible future directions. The Internet and Higher Education.

[CR5] Asarta CJ, Schmidt JR (2017). Comparing student performance in blended and traditional courses: Does prior academic achievement matter?. The Internet and Higher Education.

[CR6] Asarta CJ, Schmidt JR (2020). The effects of online and blended experience on outcomes in a blended learning environment. The Internet and Higher Education.

[CR7] Bernard RM, Borokhovski E, Schmid RF, Tamim RM, Arami PC (2014). A meta-analysis of blended learning and technology use in higher education: From the general to the applied. Journal of Computing in Higher Education.

[CR8] Bishop, J. L., & Verleger, M. A. (2013). The flipped classroom: A survey of the research. In *ASEE National Conference Proceedings, Atlanta, GA* (Vol. 30, No. 9, pp. 1–18).

[CR9] Bliuc AM, Goodyear P, Ellis RA (2007). Research focus and methodological choices in studies into students' experiences of blended learning in higher education. The Internet and Higher Education.

[CR10] Bowen WG, Chingos MM, Lack KA, Nygren TI (2014). Interactive learning online at public universities: Evidence from a six-campus randomized trial. Journal of Policy Analysis and Management.

[CR11] Brooks DC (2016). ECAR Study of Undergraduate Students and Information Technology. Research report.

[CR12] Castellanos-Reyes, D. (2020). 20 Years of the Community of Inquiry Framework. *TechTrends*.

[CR13] Chingos, M., Griffiths, R., Mulhern, C., & Spies, R. (2014). *Interactive online learning on campus: Testing MOOCs and other platforms in hybrid formats in the university system of Maryland.* New York, NY: Ithaka S+ R. Retrieved from http://www.sr.ithaka.org/publications/interactive-online-learning-on-campus/

[CR14] Garrison DR, Anderson T, Archer W (2000). Critical inquiry in a text-based environment: Computer conferencing in higher education model. The Internet and Higher Education.

[CR15] Garrison DR, Arbaugh JB (2007). Researching the community of Inquiry Framework: Review, Issues, and Future Directions. The Internet and Higher Education.

[CR16] Ginn, M., & Hammond, A. (2012). Online Education in Public Affairs: Current State and Emerging Issues. *Journal of Public Affairs Education,* *18*(2), 247–270. Retrieved from http://www.jstor.org/stable/23208653

[CR17] Halverson LR, Graham CR, Spring KJ, Drysdale JS, Henrie CR (2014). A thematic analysis of the most highly cited scholarship in the first decade of blended learning research. The Internet and Higher Education.

[CR18] Hugenholtz NI, De Croon EM, Smits PB, Van Dijk FJ, Nieuwenhuijsen K (2008). Effectiveness of e-learning in continuing medical education for occupational physicians. Occupational Medicine.

[CR19] Instructional Technology Council (ITC) (2018). 2017 distance learning survey results. *What we have learned about elearning: An overview*. Columbus, OH.

[CR20] Janus, E. S., Duhl, G. M., & Canick, S. (2014). *William Mitchell College of Law’s Hybrid Program for JD Study: Answering the Call for Innovation*.

[CR21] Joyner, D. (2018). Toward CS1 at scale: Building and testing a MOOC-for-credit candidate. L@S ’18. *Proceedings of the Fifth Annual ACM Conference on Learning at Scalehttps*.

[CR22] Lack, K. A. (2013). Current status of research on online learning in postsecondary education. *Ithaka S+ R, zuletzt geprüft am*, *3*, 2013.

[CR23] Larson DK, Sung CH (2009). Comparing student performance: Online versus blended versus face-to-face. Journal of Asynchronous Learning Networks.

[CR24] Law School Survey of Student Engagement. (2017). *Using LSSSE Data*. Retrieved from http://lssse.indiana.edu/using-lssse-data/.

[CR25] Lockee B, Moore M, Burton J (2001). Old concerns with new distance education research. Educause Quarterly.

[CR26] Major, L., Francis, G., & Tsapali, M. (2021) The effectiveness of technology-supported personalised learning in low- and middle-income countries: A meta-analysis. *British Journal of Educational Technology*. Advance online publication.

[CR27] Means, B., Toyama, Y., Murphy, R., Bakia, M., & Jones, K. (2009). Evaluation of evidence-based practices in online learning: A meta-analysis and review of online learning studies. *US Department of Education*.

[CR37] National Center for Education Statistics, U.S. Department of Education. (2016). *Digest of Education Statistics, 2015*.

[CR38] National Center for Education Statistics, U.S. Department of Education. (2021). *Digest of Education Statistics 2019.*

[CR39] National Center for Education Statistics, U.S. Department of Education. (2014). 2003–04, 2007–08, and 2011–12 National Postsecondary Student Aid Study (NPSAS:04, NPSAS:08, and NPSAS:12). See *Digest of Education Statistics 2014*, tables 311.22 and 311.32.

[CR28] Nollenberger, K. (2015). Comparing Alternative Teaching Modes in a Masters Program: Student Preferences and Perceptions. *Journal of Public Affairs Education,* *21*(1), 101–114. Retrieved from http://www.jstor.org/stable/24369707

[CR29] Oblender TE (2002). A hybrid course model-one solution to the high online drop-out rate. Learning and Leading with Technology.

[CR30] Pe´rez-Sanagustı´n M, Hilliger I, Alario-Hoyos C, Kloos CD, Rayyan S (2017). H-MOOC framework: reusing MOOCs for hybrid education. Journal of Computing in Higher Education.

[CR31] Reich J, Ruipérez-Valiente JA (2019). The MOOC pivot. Science.

[CR32] Schmid RF, Bernard RM, Borokhovski E, Tamim RM, Abrami PC, Surkes MA, Woods J (2014). The effects of technology use in postsecondary education: A meta-analysis of classroom applications. Computers & Education.

[CR33] Shea, J., Joaquin, M., & Gorzycki, M. (2015). Hybrid course design: promoting student engagement and success. *Journal of Public Affairs Education,* *21*(4), 539–556. Retrieved from http://www.jstor.org/stable/24615544

[CR34] Silver C, Rocconi L, Haeger H, Watkins L (2012). Gaining from the system: Lessons from the law school survey of student engagement about student development in law school. U. St. Thomas LJ.

[CR35] Tallent-Runnels, M., Thomas, J., Lan, W., Cooper, S., Ahern, T., Shaw, S., & Liu, X. (2006). Teaching courses online: A review of the research. *Review of Educational Research,* *76*(1), 93–135. Retrieved from http://www.jstor.org/stable/3700584

[CR36] Twigg CA (2003). Improving learning and reducing costs: New models for online learning. Educause Review.

[CR40] U.S. Department of Education. (2020). *What Works Clearinghouse Standards Handbook, Version 4.1*. Washington, DC: U.S. Department of Education, Institute of Education Sciences, National Center for Education Evaluation and Regional Assistance.

[CR41] UNESCO. (2020). Education from disruption to recover. https://en.unesco.org/covid19/educationresponse

[CR43] Ward B (2004). The best of both worlds: A hybrid statistics course. Journal of Statistics Education.

[CR44] Wilke, D., & Vinton, L. (2006). Evaluation of the first web-based advanced standing MSW program. Journal of Social Work Education, 42(3), 607–620. Retrieved from http://www.jstor.org/stable/23044197.

[CR45] Xu D, Jaggars SS (2014). Performance gaps between online and face-to-face courses: Differences across types of students and academic subject areas. The Journal of Higher Education.

[CR46] York, R. (2008). Comparing three modes of instruction in a graduate social work program. Journal of Social Work Education, 44(2), 157–172. Retrieved from http://www.jstor.org/stable/23044285.

[CR42] Yuping Wang, Xibin Han, & Juan Yang. (2015). Revisiting the Blended Learning Literature: Using a Complex Adaptive Systems Framework. Journal of Educational Technology & Society,18(2), 380–393. Retrieved from http://www.jstor.org/stable/jeductechsoci.18.2.380.

[CR47] Zheng, Q., Chen, L., & Burgos, D. (2018). Certificate authentication and credit system of MOOCs in China. *The development of MOOCs in China* (pp. 261–276). Springer Singapore.

